# Association of Unexpected Newborn Deaths With Changes in Obstetric and Neonatal Process of Care

**DOI:** 10.1001/jamanetworkopen.2020.24589

**Published:** 2020-12-07

**Authors:** Dan Han, Aayush Khadka, Margaret McConnell, Jessica Cohen

**Affiliations:** 1 Lee Kuan Yew School of Public Policy, National University of Singapore, Singapore; 2 Harvard T.H. Chan School of Public Health, Boston, Massachusetts

## Abstract

**Importance:**

The death of a healthy term infant may signal patient safety and quality issues. Various initiatives aim to encourage clinicians to learn from these events, but little evidence exists regarding how exposure to an unexpected newborn death may alter clinician practice.

**Objective:**

To examine the association between an unexpected newborn death and changes in obstetric and newborn procedures that may be used in response to potential fetal distress or newborn complications.

**Design, Setting, and Participants:**

This cross-sectional study used difference-in-differences analysis of 2011 to 2017 US vital statistics data from 477 US counties experiencing an unexpected newborn death during the study period. All in-hospital live births in the 477 counties during the study period were included. Data were analyzed from September 2019 to September 2020.

**Exposures:**

The death of an infant aged 0 to 7 days following an unremarkable pregnancy owing to causes other than birth defects, accidents/assaults, or sudden infant death syndrome.

**Main Outcomes and Measures:**

Primary outcomes included binary variables capturing intervention in labor/delivery (induction, augmentation, cesarean delivery, forceps/vacuum) and procedures to avert and mitigate newborn complications (assisted ventilation, surfactant replacement therapy, antibiotics for suspected sepsis, neonatal intensive care unit admission).

**Results:**

The main sample included 5.72 million births (2.54 million during preexposure time). Mean (SD) maternal age was 27.3 (5.8) years; 67% of mothers were White, and 12% were Black. Associations varied across the 4 estimated models. Following an unexpected newborn death, there was no significant increase in the probability of cesarean delivery in the full sample model (0.28 percentage points [pp]; 95% CI, −0.01 to 0.57 pp), but a significant increase in the other 3 models, with values ranging from 0.55 pp (95% CI, 0.21 to 0.88 pp) in the full sample model with matching to 0.66 pp (95% CI, 0.13 to 1.19 pp) in the 1-hospital county subsample with matching. There was a significant increase in the probability of newborn assisted ventilation in the full sample model with matching (0.46 pp; 95% CI, 0.08 to 0.83 pp), but no significant increase in the other 3 models, with estimates ranging from 0.33 pp (95% CI, −0.04 to 0.71 pp) to 0.69 pp (95% CI, −0.02 to 1.40 pp). An unexpected newborn death was not associated with a significant increase in antibiotic use in the full sample models (without matching: 0.19 pp; 95% CI, −0.00 to 0.39 pp; with matching: 0.22 pp; 95% CI: −0.02 to 0.46 pp), but was associated with a significant increase in both of the 1-hospital county subsample models (without matching: 0.38 pp; 95% CI, 0.02 to 0.73 pp; with matching: 0.39 pp; 95% CI, 0.01 to 0.77 pp).

**Conclusions and Relevance:**

In some study models, an unexpected newborn death was associated with statistically significant increases in subsequent use of procedures to avert and mitigate fetal distress and newborn complications, which could reflect increases in identifying and proactively addressing serious potential complications or increased clinician caution applied across all cases. Future research should address whether these changes affect patient outcomes.

## Key Points

**Question:**

Was the occurrence of an unexpected newborn death associated with changes in obstetric and neonatal process of care?

**Findings:**

In this cross-sectional study using difference-in-differences analysis, an unexpected newborn death was associated with a modest increase in the subsequent use of cesarean delivery, newborn assisted ventilation, and antibiotic use for suspected sepsis, as well as an increase in neonatal intensive care unit admission in subgroup analysis.

**Meaning:**

Unexpected newborn deaths were associated with increases in subsequent use of procedures to avert and mitigate fetal distress and newborn complications.

## Teaser

This cross sectional study investigates whether occurrence of an unexpected newborn death is associated with changes in obstetric and neonatal process of care.

## Introduction

More than 7000 full-term infants die each year in the United States, equivalent to roughly 2.2 deaths per 1000 term births.^1^ About one-fifth of these deaths occur within the first week of birth.^1^ Although sudden infant death syndrome and congenital malformation represent the majority of term newborn deaths,^1^ some newborn deaths are preventable and may be attributable to inadequate quality of care during childbirth and the early neonatal period.^2^ Increasing research documents wide variation in use of obstetric and neonatal procedures and term newborn complications across hospitals in the US, suggesting room for quality improvement and standardization.^3,4,5,6^

Various policy initiatives have required or encouraged retrospective examination of unexpected and potentially avertible newborn deaths with the aim of improving childbirth safety and quality of routine obstetric and newborn care. The Joint Commission requires accredited hospitals to review and develop a proper response to the unanticipated death of a full-term infant, and the National Quality Forum includes the death of a neonate associated with labor or delivery in a low-risk pregnancy as a serious adverse event to be reported by states.^7,8,9^ The Fetal and Infant Mortality Review program reviews selected cases of fetal and infant deaths to identify weaknesses of medical and nonmedical systems.^10^

Despite efforts encouraging learning from unexpected newborn deaths, little evidence exists about whether exposure to unexpected newborn death leads to changes in obstetric and newborn care practice. Studies examining clinician behavior in other medical settings find that learning about the recent occurrence of a severe adverse event changes clinician decision-making in ways that can have important implications for patient outcomes.^11,12,13,14^ Related literature on comparative quality of care reporting suggests that public reporting can trigger clinician responses that improve quality but also can generate unintended consequences.^15,16^

In this study, we investigated whether the death of an otherwise healthy term newborn following an unremarkable pregnancy was associated with subsequent use of obstetric and newborn procedures. After an unexpected newborn death, clinicians may become more responsive to potential signs of fetal or newborn distress and complications, or their decisions about when to intervene could become more conservative. These responses could manifest through changes in delivery-related obstetric procedures and/or newborn procedures. We leveraged the variation in the timing of a death across counties to examine the association between unexpected deaths and obstetric and neonatal procedures using the US vital statistics data.

## Methods

### Data

This study followed the Strengthening the Reporting of Observational Studies in Epidemiology (STROBE) reporting guideline.^17^ This study was based on deidentified secondary data and deemed exempt by the Institutional Review Board at the Harvard T.H. Chan School of Public Health. Our primary data came from the restricted-use US vital statistics data from 2011 to 2017, which included data from birth certificates of all live births in the US and data from infant death certificates linked to their birth certificates. We excluded 14 states (18% of all births) that had not fully adopted the 2003 version of the birth certificate by 2011. To account for local socioeconomic status and health care resources, we merged in data from the US Census and the Centers for Medicare & Medicaid Services (eTable 1 in the Supplement). Data were analyzed from September 2019 to September 2020.

### Definition of Exposure and Sample Selection

The exposure of interest was the occurrence of an unexpected newborn death at the county level. “Unexpected newborn death” was defined as the death of an infant aged 0 to 7 days following an unremarkable pregnancy owing to causes other than birth defects, accidents, assaults, or sudden infant death syndrome. An unremarkable pregnancy was defined as a singleton pregnancy to a woman aged 18 to 40 years who gave birth to a newborn with birthweight greater than 2500 g, gestational age between 37 and 42 weeks, and no congenital anomalies. We focused on deaths among infants that were born in hospitals and died in acute care settings. In all, 737 counties in our data set experienced at least 1 unexpected newborn death between the first quarter (Q1) of 2011 and Q4 of 2017.

The exposure was defined as the first incidence of unexpected newborn death within a county during the study period. To ensure enough preexposure and postexposure data and sufficient sample size, we restricted the exposure group to counties whose first incidence occurred between Q1 of 2012 and Q1 of 2016. We excluded counties with fewer than 25 average quarterly births and counties with fewer than 25 births in any given quarter, as data for these counties may be subject to greater noise and measurement error. Two counties that appeared to be outliers (with >10 deaths during the study period) were dropped. Within the remaining sample, roughly 80% of counties did not experience a second death during the study period or did so at least a year after the first death. Our final sample was constructed based on all in-hospital births in 477 ever-exposed counties (eTable 2 in the Supplement).

### Outcomes and Covariates

We analyzed outcomes related to processes of care during delivery and the early newborn period, focusing on procedures that could be indicated based on signs of fetal or newborn distress or complications and that are captured in national vital statistics data. Process measures related to labor and delivery included binary variables for induction, augmentation, cesarean delivery, and delivery using forceps/vacuum. Newborn procedures included binary variables for assisted ventilation immediately after delivery or for more than 6 hours, receipt of surfactant replacement therapy, receipt of antibiotics for suspected sepsis, and admission to a neonatal intensive care unit (NICU). We stratified the analysis of cesarean delivery by whether the delivery was scheduled or unplanned and whether the birth was considered a low-risk first birth (eMethods in the Supplement).^18^ Since some of the examined labor and delivery procedures can have downstream impacts on maternal outcomes, we explored the association between the exposure and maternal complications/procedures in eTable 8 in the Supplement, including the presence of third- or fourth-degree laceration, ruptured uterus, unplanned hysterectomy, blood transfusion, and admission to an intensive care unit.

We included the following birth-level covariates: mother’s race/ethnicity, marital status, age, education, nulliparous/multiparous, singleton/multiple, health insurance type, smoking status during pregnancy, maternal infections during pregnancy, obesity, late/no antenatal care, history of cesarean delivery, prepregnancy/gestational diabetes, prepregnancy/gestational hypertension, breech presentation, sex of the infant, birthweight less than 2500 g, birthweight greater than 4000 g, preterm birth, and any congenital anomalies. County-level covariates included yearly measures of poverty rate, median household income, uninsured rate, number of hospitals offering obstetric care, number of hospitals with a NICU, number of obstetricians per 1000 women aged 15 to 44 years, and number of primary care physicians per 1000 individuals (eTable 1 in the Supplement).

### Statistical Analysis

We used a stacked difference-in-differences (DD) design,^19,20,21^ in which counties experiencing a newborn death currently (the exposure group, or current-exposed) were compared with future-exposed counties during the period when the future-exposed counties had yet to experience a death (eMethods in the Supplement). We used the future-exposed counties as the comparison group because the current-exposed and future-exposed counties were much more similar to each other across maternal and birth characteristics and birth volume than to the never-exposed counties (eTable 3 in the Supplement). We considered changes in the outcomes over the 4 quarters before and after a newborn death (ie, from −4 to +4). For counties experiencing a death in quarter *k*, we paired them with a comparison group comprising counties experiencing a death over the period between *k *+ 4 + 1 and *k *+ 4 + 6 (eFigure 1 and eTable 4 in the Supplement). Each pair formed a DD cohort defined by *k*; we pooled together all cohorts for the regression analysis. The identification assumption was that absent exposure, outcomes in the exposure and comparison counties would have moved in parallel, conditional on covariates. A second assumption was that the timing of the event was unpredictable so there was no anticipatory behavior right before the event.

We estimated 2 birth-level linear regression models with fixed effects for county-cohort and year-quarter and adjusted for birth characteristics and time-varying county characteristics. The first model interacted a binary variable for current-exposed counties with a series of quarter-to-event dummy variables; we presented the estimated coefficients from these models graphically. In the second model, we estimated the average differential change by replacing the quarter-to-event dummies with a binary variable for all quarters postexposure.

We also conducted a subgroup analysis among the 245 counties with only 1 hospital offering obstetric care throughout 2011 to 2017 for 2 reasons. First, this analysis focused on only the clinicians in hospitals that experienced the exposure, rather than clinicians in unexposed hospitals in the same county as the exposed hospitals. Second, this analysis reduced potential confounding from changes in patient utilization patterns following the unexpected newborn death.

In our study design, counties with higher birth volumes were more likely to experience a death earlier in time and therefore were disproportionately represented in the exposure group. We performed coarsened exact matching (CEM) to improve comparability between the exposure and comparison groups over birth volume and county health care resources (eMethods in the Supplement).^22^ The CEM-matched data retained just above two-thirds of the observations in the exposure group and slightly more than half of the observations in the comparison group (eTable 5 in the Supplement). We repeated the analysis within the full set of CEM-matched data and the subset of matched data for counties with 1 hospital.

Standard errors were clustered at the county level to account for correlated data within counties. The threshold for statistical significance was set at 2-tailed *P* < .05. All analyses were performed using Stata version 15 (StataCorp).^23^

### Sensitivity Analysis

We conducted 2 sensitivity analyses. First, since newborn deaths occurring further from the delivery date may be less likely to occur in the hospital used for delivery, we performed an analysis restricting to unexpected deaths of newborns aged 0 to 2 days. Second, for individual outcomes within an outcome domain (ie, labor/delivery procedures, newborn procedures, and maternal complications/procedures), as a robustness check we performed the Benjamini-Hochberg procedure to control for a false-discovery rate of 0.1.^24,25^

## Results

### Summary Statistics

Of the 402 unexpected newborn deaths included in the analysis, 39.3% occurred within the first 24 hours, 36.2% were vaginal deliveries, 23.8% were unplanned cesarean deliveries, 40% were scheduled cesarean deliveries, and most were related to low oxygen levels in the newborn (Table 1). Of the 2 539 031 births observed during preexposure time, the mean (SD) maternal age was 27.3 (5.8), 67% of mothers were White, 12% were Black, 47.5% had Medicaid, 32.2% were nulliparous, and 10.7% were preterm (Table 2).

**Table 1.** Characteristics of Newborn Deaths Meeting Selection Criteria and Included in Analysis^a^

Characteristics	No. (%)	
	All newborn deaths meeting selection criteria (n = 2698)^b^	Newborn deaths included in the analysis (n = 402)^c^
Age at death, d		
0	972 (36.0)	158 (39.3)
1-3	968 (35.9)	132 (32.8)
4-7	758 (28.1)	112 (27.9)
Mode of delivery^d^		
Vaginal delivery	1050 (39.2)	145 (36.2)
Cesarean delivery		
Unplanned	623 (23.3)	95 (23.8)
Scheduled	1004 (37.5)	160 (40.0)
Birth order		
First birth	1081 (40.1)	161 (40.0)
Top causes of deaths^e^		
P91.6 Hypoxic ischemic encephalopathy of newborn	397 (14.7)	63 (15.7)
P21.9 Birth asphyxia unspecified	178 (6.6)	36 (9.0)
P02.1 Newborn affected by other forms of placental separation and hemorrhage	150 (5.6)	27 (6.7)
P24.0 Neonatal aspiration of meconium	119 (4.4)	14 (3.5)
P20.9 Intrauterine hypoxia unspecified	116 (4.3)	21 (5.2)
P36.9 Bacterial sepsis of newborn unspecified	100 (3.7)	15 (3.7)
P29.1 Neonatal cardiac dysrhythmia	77 (2.9)	16 (4.0)

^a^ We excluded 14 states using the old birth certificate: Alaska, Alabama, Arkansas, Arizona, Connecticut, Hawaii, Massachusetts, Maine, Minnesota, Mississippi, New Jersey, Rhode Island, Virginia, and West Virginia.

^b^ Column includes all newborn deaths in 2011-2017 meeting the selection criteria for “unexpected newborn death,” ie, the death of an infant aged 0-7 days in a hospital following an unremarkable pregnancy due to causes other than birth defects, accidents, assaults, or sudden infant death syndrome.

^c^ Column shows the subset of unexpected newborn deaths included in the final analytic data set.

^d^ Mode of delivery was missing for 2 of 402 included deaths.

^e^ Unspecified diagnoses not listed.

**Table 2.** Maternal/Birth and County Characteristics of Current- vs Future-Exposed Counties During Preexposure Time^a^^,^^b^

Characteristic	No. (%)		
**Total**	**Current-exposed counties**	**Future-exposed counties**
**Panel A: Individual maternal/birth **			
No.	2 539 031	840 518	1 698 513
Race/ethnicity			
White	1 700 532 (67.0)	540 456 (64.3)	1 160 076 (68.3)
Black	305 712 (12.0)	113 638 (13.5)	192 074 (11.3)
Hispanic	419 918 (16.5)	147 299 (17.5)	272 619 (16.1)
Other	112 869 (4.4)	39 125 (4.7)	73 744 (4.3)
Insurance coverage^c^			
Medicaid	1 204 880 (47.5)	371 953 (44.3)	832 927 (49.0)
Private insurance	1 140 401 (44.9)	405 371 (48.2)	735 030 (43.3)
Uninsured	74 544 (2.9)	24 083 (2.9)	50 461 (3.0)
Highest education			
Less than high school	422 694 (16.6)	134 187 (16.0)	288 507 (17.0)
High school or some college	1 485 934 (58.5)	472 170 (56.2)	1 013 764 (59.7)
College degree or above	630 403 (24.8)	234 161 (27.9)	396 242 (23.3)
Maternal age, mean (SD), y	27.3 (5.8)	27.6 (5.8)	27.2 (5.8)
Married	1 462 448 (57.6)	500 471 (59.5)	961 977 (56.6)
Nulliparous	816 864 (32.2)	269 432 (32.1)	547 432 (32.2)
Multiple gestations	76 551 (3.0)	28 483 (3.4)	48 068 (2.8)
Smoking during pregnancy	328 150 (12.9)	95 846 (11.4)	232 304 (13.7)
Late or no antenatal care	144 493 (5.7)	47 563 (5.7)	96 930 (5.7)
Obesity	649 972 (25.6)	206 615 (24.6)	443 357 (26.1)
Maternal infection	70 184 (2.8)	23 521 (2.8)	46 663 (2.7)
Chronic/gestational			
Diabetes	153 070 (6.0)	51 321 (6.1)	101 749 (6.0)
Hypertension	171 021 (6.7)	58 658 (7.0)	112 363 (6.6)
Previous Cesarean delivery	366 852 (14.4)	121 190 (14.4)	245 662 (14.5)
Breech presentation	91 286 (3.6)	31 197 (3.7)	60 089 (3.5)
Birth weight, mean (SD), g	3293.1 (568.4)	3281.8 (585.3)	3298.6 (559.7)
Preterm birth	271 175 (10.7)	94 854 (11.3)	176 321 (10.4)
Female infant	1 238 718 (48.8)	409 997 (48.8)	828 721 (48.8)
Congenital anomalies	9010 (0.4)	3193 (0.4)	5817 (0.3)
Mode of this delivery: Cesarean delivery	816 875 (32.2)	269 678 (32.1)	547 197 (32.2)
**Panel B: County -year measures, mean (SD)**			
No.	3064^d^	659^d^	2405^d^
Births per quarter^e^	402.6 (506.0)	589.9 (717.6)	351.2 (416.0)
Population (thousands)	129.3 (160.8)	178.7 (206.1)	115.7 (143.0)
Nonmetropolitan (%)	45.4 (49.8)	40.4 (49.1)	46.7 (49.9)
No. of hospitals with obstetric services	1.5 (1.1)	1.8 (1.3)	1.5 (0.9)
% Counties without NICU	73.2 (44.3)	64.0 (48.0)	75.7 (42.9)
OB/GYN per 1000 female aged 15-44 y	0.5 (0.4)	0.6 (0.5)	0.5 (0.3)
Primary care physicians per 1000 individuals	0.9 (0.6)	1.0 (0.8)	0.9 (0.5)
Median household income, 2017 $	50.3 (11.9)	52.0 (13.3)	49.8 (11.5)
% Population in poverty	16.6 (5.7)	16.2 (5.6)	16.7 (5.7)
Uninsured rate among individuals aged 18-64 y	15.9 (5.3)	16.0 (5.3)	15.8 (5.4)

Abbreviations: NICU, neonatal intensive care unit; OB/GYN, obstetrician/gynecologist.

^a^ Included data for quarter-to-event −4 through −1 (1-year period).

^b^ We excluded 14 states using the old birth certificate: Alaska, Alabama, Arkansas, Arizona, Connecticut, Hawaii, Massachusetts, Maine, Minnesota, Mississippi, New Jersey, Rhode Island, Virginia, and West Virginia.

^c^ Numbers do not add up to 100% owing to other/unknown insurance type.

^d^ The No. for county characteristics reported in Panel B is the number of county-year observations. It is larger than the number of individual counties because the quarter-to-event time did not necessarily align with the calendar year, so some partial year data was used.

^e^ Quarterly birth volume averaging over the 1-year preexposure period.

Individual- and county-level characteristics were generally comparable between the current-exposed and future-exposed counties, though some differences existed (Table 2). Current-exposed counties were on average larger, with higher birth volume, and less likely to be in rural areas. Pregnant women in current-exposed counties were somewhat more likely to be Black or Hispanic, more likely to be married, have private insurance, and have higher levels of education. They were slightly less likely to have obesity or to smoke during pregnancy.

### Regression Results

**Figure 1.**
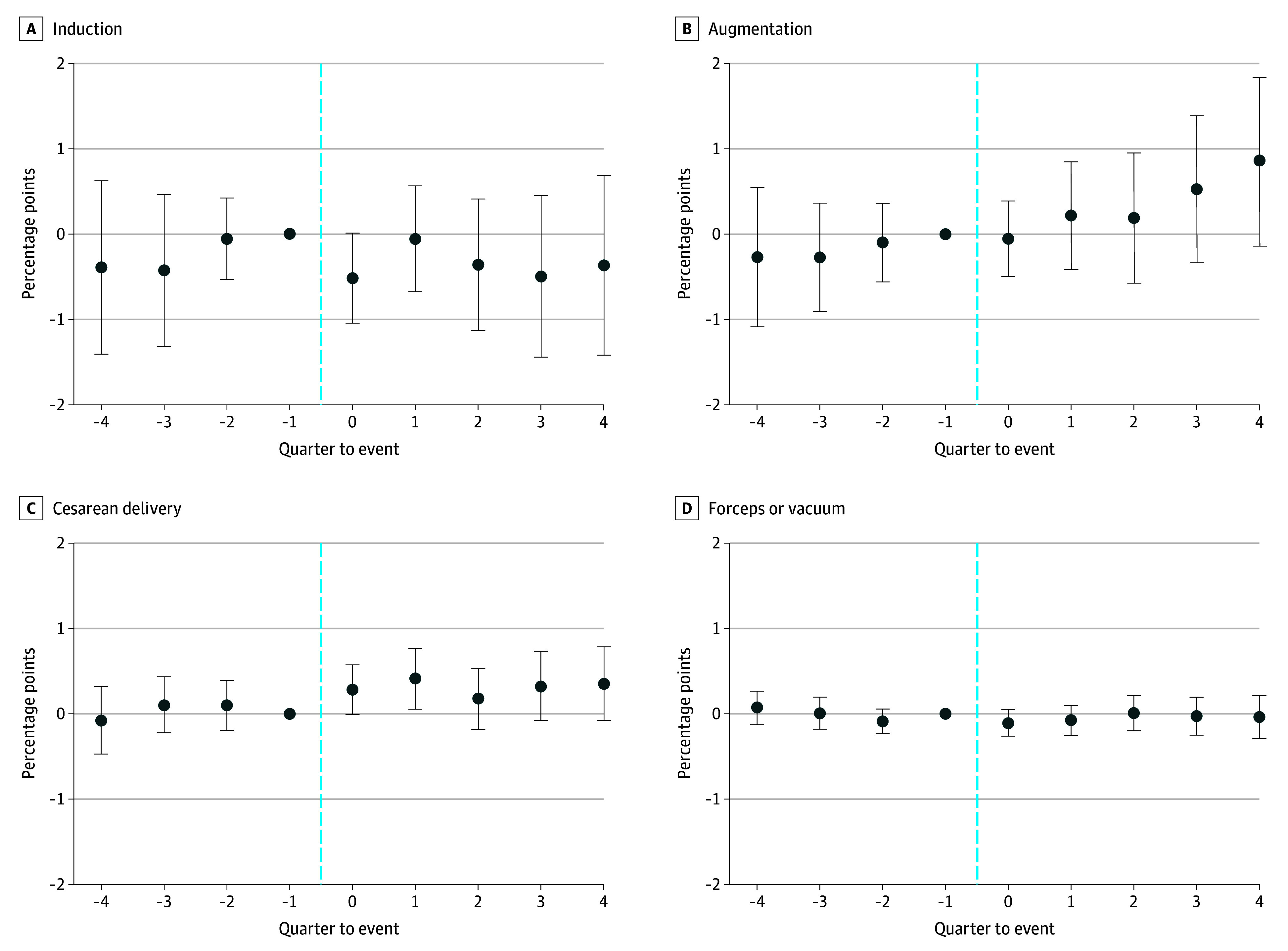
Use of Obstetric Procedures Following an Unexpected Newborn Death Quarter-to-event coefficient estimates represent changes in percentage points in the outcome (points) and 95% confidence interval (error bars); coefficients were estimated relative to time −1 and the confidence intervals were calculated using standard errors clustered at the county level. The *P* value for the joint significance test of the preexposure coefficients was .75 for induction, .56 for cesarean delivery, .33 for forceps/vacuum, and .86 for augmentation.

**Figure 2.**
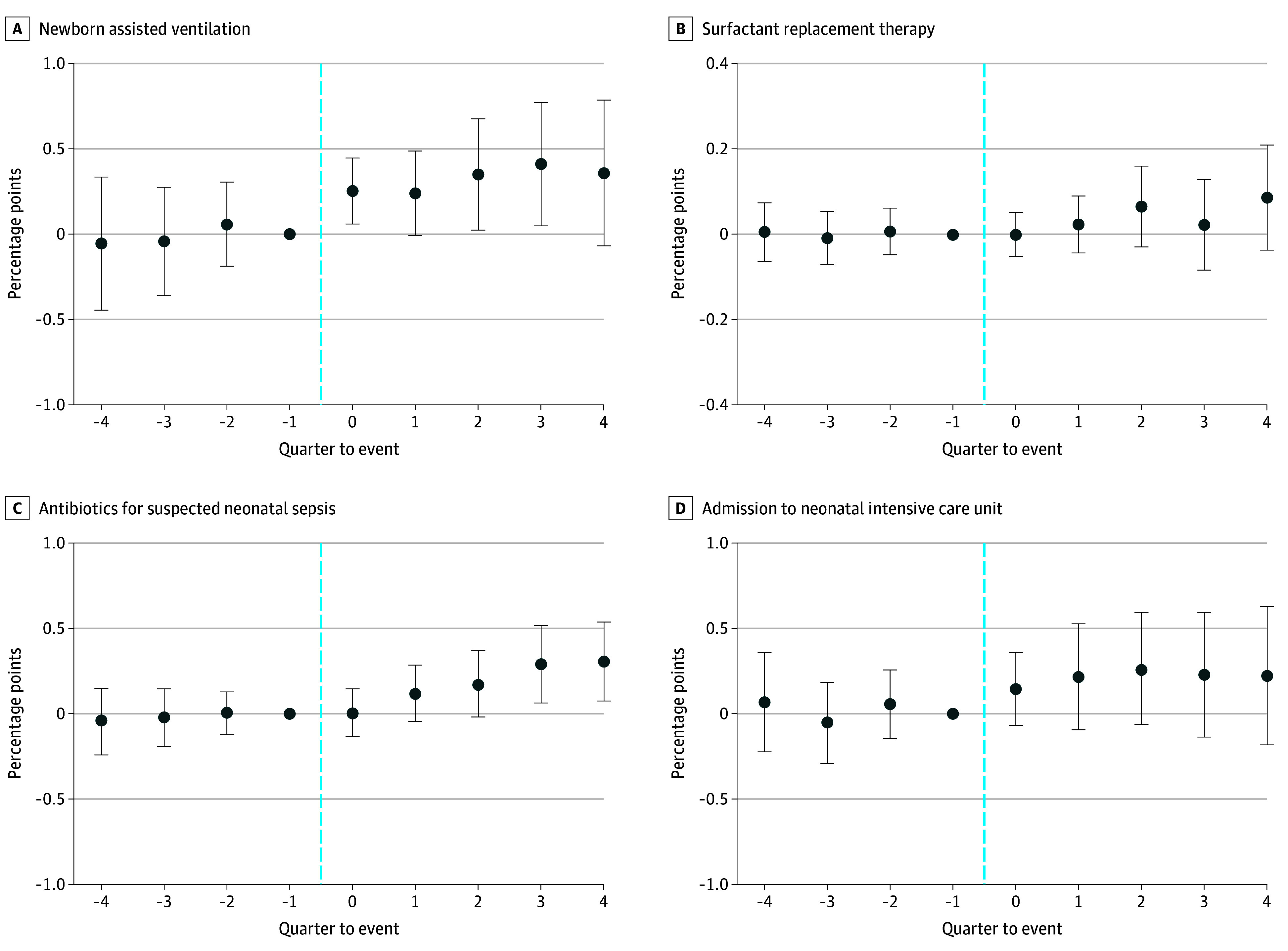
Use of Newborn Procedures Following an Unexpected Newborn Death Quarter-to-event coefficient estimates represent changes in percentage points in the outcome (points) and 95% confidence interval (error bars); coefficients were estimated relative to time −1 and the confidence intervals were calculated using standard errors clustered at the county level. The *P* value for the joint significance test of the preexposure coefficients .71 for assisted ventilation, .95 for surfactant therapy, .95 for antibiotics for suspected sepsis, and .56 for admitted to neonatal intensive care unit.

The estimated coefficients on preexposure quarter-to-event −4 through −2 were not significantly different from zero across all outcomes, suggesting that trends in the outcomes before the event were similar in the current- and future-exposed countries (Figure 1 and Figure 2). The *P* values of the *F* test of preexposure coefficients jointly equal to zero was .75 for induction, .56 for cesarean delivery, .33 for forceps/vacuum, .86 for augmentation, .71 for assisted ventilation, .95 for surfactant therapy, .95 for antibiotics use for suspected sepsis, and .56 for NICU admission.

Postexposure, there was a modest increase in cesarean delivery in the first quarter following an unexpected newborn death (ie, quarter-to-event = 1; Figure 1), and statistically significant associations were found in 3 of the 4 models estimated (Table 3). An unexpected newborn death was not associated with a significant increase in the probability of cesarean delivery in the full sample model (0.28 percentage points [pp]; 95% CI, −0.01 to 0.57 pp; *P* = .06; 0.9% of sample mean; Table 3, panel A), but was associated with a significant increase in cesarean delivery in the other 3 models, with values ranging from 0.55 pp in the full sample model with matching (95% CI, 0.21 to 0.88 pp; *P* < .01; 1.7% of sample mean; Table 3, panel C) to 0.66 pp in the 1-hospital county subsample model with matching (95% CI, 0.13 to 1.19 pp; *P* = .02; 2.1% of sample mean; Table 3, panel D). While augmentation appeared to increase gradually following an unexpected newborn death (Figure 1), the estimate was statistically insignificant in the full sample model and inconsistent across models. We observed no changes in the probabilities of induction or forceps/vacuum.

**Table 3.** Postexposure Change in Obstetric and Newborn Procedures Among Current-Exposed Counties Relative to Future-Exposed Counties^a^^,^^b^^,^^c^

	Induction	Cesarean delivery	Forceps or vacuum	Augmentation	Assisted ventilation (immediate or >6 h)	Surfactant replacement therapy	Antibiotics for suspected neonatal sepsis	Admission to NICU
**Panel A: Full set of counties**								
Point estimate (95% CI)	−0.14 (−0.99 to 0.70)	0.28 (−0.01 to 0.57)	−0.05 (−0.20 to 0.11)	0.50 (−0.25 to 1.25)	0.33 (−0.04 to 0.71)	0.04 (−0.04 to 0.12)	0.19 (−0.00 to 0.39)	0.20 (−0.14 to 0.53)
*P* value	.74	.06	.57	.19	.08	.36	.05	.24
Mean of dep variable	28.14	32.16	3.45	21.43	3.83	0.50	2.26	6.86
Observations	5 723 182	5 723 201	5 723 204	5 723 182	5 707 555	5 707 555	5 707 555	5 707 555
**Panel B: Counties with 1 hospital**								
Point estimate (95% CI)	−0.04 (−2.42 to 2.34)	0.60 (0.09 to 1.11)^d^	−0.24 (−0.65 to 0.17)	−0.43 (−2.03 to 1.17)	0.69 (−0.02 to 1.40)^d^	0.02 (−0.14 to 0.18)	0.38 (0.02 to 0.73)^d^	0.62 (−0.03 to 1.27)^d^
*P* value	.97	.02	.25	.60	.06	.82	.04	.06
Mean of dep variable	29.19	30.83	4.09	21.82	3.27	0.43	2.04	5.30
Observations	1 862 825	1 862 798	1 862 799	1 862 825	1 855 717	1 855 717	1 855 717	1 855 717
**Panel C: Full set of counties, coarsened exact matching**								
Point estimate (95% CI)	0.15 (−0.96 to 1.27)	0.55 (0.21 to 0.88)^d^	−0.15 (−0.34 to 0.05)	0.70 (−0.20 to 1.59)	0.46 (0.08 to 0.83)^d^	−0.01 (−0.09 to 0.08)	0.22 (−0.02 to 0.46)	0.09 (−0.37 to 0.54)
*P* value	.78	<.01	.14	.13	.02	.89	.07	.71
Mean of dep variable	27.81	32.21	3.58	21.45	3.73	0.49	2.32	7.02
Observations	3 429 351	3 429 380	3 429 380	3 429 351	3 423 433	3 423 433	3 423 433	3 423 433
**Panel D: Counties with 1 hospital, coarsened exact matching**								
Point estimate (95% CI)	0.16 (−2.24 to 2.55)	0.66 (0.13 to 1.19)^d^	−0.26 (−0.69 to 0.16)	−0.48 (−2.19 to 1.23)	0.67 (−0.06 to 1.39)^d^	−0.02 (−0.17 to 0.13)	0.39 (0.01 to 0.77)^d^	0.70 (−0.05 to 1.45)^d^
*P* value	.90	.02	.22	.58	.07	.78	.04	.07
Mean of dep variable	28.43	30.88	4.04	21.26	3.21	0.39	2.03	5.15
Observations	1 281 559	1 281 676	1 281 676	1 281 559	1 279 243	1 279 243	1 279 243	1 279 243

Abbreviation: NICU, neonatal intensive care unit.

^a^ We excluded 14 states using the old birth certificate: Alaska, Alabama, Arkansas, Arizona, Connecticut, Hawaii, Massachusetts, Maine, Minnesota, Mississippi, New Jersey, Rhode Island, Virginia, and West Virginia.

^b^ All coefficients were estimated using linear regressions with a binary dependent variable and thus represent percentage point changes in the outcome.

^c^ All confidence intervals were calculated using standard errors clustered at the county level.

^d^ Statistically significant after controlling for a false positive rate of 0.1 using the Benjamini-Hochberg procedure.

Turning to newborn procedures, there was an immediate increase in newborn assisted ventilation and a gradual increase in antibiotic use for suspected sepsis (Figure 2), with the estimates statistically significant in some, but not all, of the models (Table 3). After an unexpected newborn death, there was a statistically significant increase in the probability of assisted ventilation in the full sample model with matching (0.46 pp; 95% CI, 0.08 to 0.83 pp; *P* = .02; 12.3% of sample mean; Table 3, panel C), but no significant increase in assisted ventilation in the other 3 models, with these estimates ranging from 0.33 pp in the full sample model (95% CI, −0.04 to 0.71 pp; *P* = .08; 8.6% of sample mean; Table 3, panel A) to 0.69 pp in the 1-hospital county subsample model (95% CI, −0.02 to 1.40; *P* = .06; 21.1% of sample mean; Table 3, panel B). There was no significant increase in the probability of antibiotic use in the full sample models, without matching (0.19 pp; 95% CI, −0.00 to 0.39 pp; *P* = .05; 8.4% of sample mean; Table 3, panel A) or with matching (0.22 pp; 95% CI, −0.02 to 0.46 pp; *P* = .07; 9.5% of sample mean; Table 3, panel C), but a significant increase in antibiotic use in the 1-hospital county subsample models, without matching (0.38 pp; 95% CI, 0.02 to 0.73 pp; *P* = .04; 18.6% of sample mean; Table 3, panel B) and with matching (0.39 pp; 95% CI, 0.01 to 0.77 pp; *P* = .04; 19.2% of sample mean; Table 3, panel D).

We estimated statistically nonsignificant but practically meaningful postexposure changes in NICU admission in the 1-hospital county subsample, without matching (0.62 pp; 95% CI, −0.03 to 1.27 pp; *P* = .06; 11.7% of sample mean; Table 3 panel B) and with matching (0.70 pp; 95% CI, −0.05 to 1.45 pp; *P* = .07; 13.6% of sample mean; Table 3 panel D). The estimates for surfactant therapy were small, statistically insignificant, and inconsistent across all models.

The increase in cesarean delivery was more pronounced for low-risk first-births (eTable 6 in the Supplement). Results based on newborn deaths within 0 to 2 days were largely consistent with the main results (eTable 7 in the Supplement). Our results were generally robust to controlling for a false-discovery rate of 0.1.

As to downstream maternal procedures/complications, most estimates were practically small except for the estimated reduction in third- and fourth-degree perineal laceration, but it was statistically insignificant across all 4 models (eTable 8 in the Supplement).

## Discussion

Unexpected newborn deaths are sentinel events that may signal a need for clinician practice reviews and quality improvement initiatives. We found that, following an unexpected newborn death, some models indicated that clinicians increased use of some obstetric and newborn procedures meant to respond to signs of fetal or newborn distress and complications, including cesarean delivery, newborn assisted ventilation, and antibiotics use for suspected sepsis. In 1-hospital counties, estimated changes were larger, possibly because responses in multihospital counties were diluted by hospitals that did not experience the unexpected death.

The estimated association between an unexpected newborn death and cesarean delivery, while modest and statistically nonsignificant in 1 of the 4 models, was comparable to estimates of changes in cesarean delivery use associated with policy-relevant factors such as payment differentials and clinician income shocks due to declining fertility rates in other observational studies.^26,27,28^ Our estimates for neonatal procedures were also comparable with or larger than those associated with factors such as incentives to fill empty NICU beds and delivering at a hospital with a high rate of cesarean delivery.^29,30^

It is possible that clinicians in this study became more cautious following an unexpected newborn death in their county and may therefore have performed more obstetric and neonatal procedures. For example, at least one-third of deaths included in this analysis were associated with low oxygen levels in newborns. Despite limited evidence about the effect of cesarean delivery on birth asphyxia,^31,32^ clinicians may initiate cesarean delivery to avoid prolonged labor—a risk factor for birth asphyxia.^33^ Litigation pressure could lead to further precaution and possibly “defensive” cesarean deliveries, as by one estimate the most common diagnoses involved in obstetrics malpractice lawsuits was hypoxic-ischemic encephalopathy and severe birth asphyxia.^34,35^ Another important possibility was that clinicians learned about quality gaps, such as misjudgment of situations that should have required cesarean delivery.

Similarly, increases in newborn assisted ventilation and antibiotics use for suspected sepsis may be due to greater caution and/or improved care on the part of clinicians. It is notable that some of the biggest observed increases in neonatal procedures were surrounding treatment of suspected sepsis, although sepsis-related complications were only relevant for 3.7% of the unexpected newborn deaths. Recent work has documented highly variable antibiotic use across NICUs but low correlation with actual infection,^4^ suggesting that use of antibiotics may be influenced by clinician discretion and practice style.^36^

These results may help inform current efforts to improve maternal and newborn death review and quality of care. Recent evidence has highlighted the challenges in measuring sentinel events in obstetric and perinatal care that accurately identify potential quality concerns.^37^ Based on the estimates from some of our models, we found that a sentinel event like the death of an otherwise healthy newborn is associated with changes in processes of care for labor/delivery and for newborns, suggesting that these events present new information on performance for clinicians. However, clinicians may learn from the event and improve care processes or react defensively and increase interventions for all deliveries. Standardized procedures to review adverse events, share data, and monitor changes could help understand clinicians’ response and the implications for patient outcomes.^38,39^

### Limitations

This study had several limitations. First, our data did not allow for evaluating whether overall quality of care or clinical outcomes improved following an unexpected newborn death. Second, given the absence of hospital and clinician identifiers in the data, our estimates mainly captured the local aggregate response, which did not represent any particular hospital’s or clinician’s practice and may be diluted by unexposed hospitals in the same county. This concern was ameliorated somewhat by the 1-hospital county subgroup analysis, which showed broadly consistent findings. Third, one-third of the unexpected deaths occurred among newborns transferred out of their birth county. To the extent that these transfers weakened the response of the transferring clinicians because they may not know of the death or they may not attribute the death to their own actions, our estimates may have underestimated changes in clinician practice. Fourth, stillbirths were not examined because data on fresh vs macerated stillbirths was inconsistently reported in our data and because stillbirths occurred too frequently to enable our analytical setup. Finally, given the imposed sample restrictions, our analysis may not be representative of small, rural counties with low population density.

## Conclusions

We found that , in some models, an unexpected newborn death at the county level was associated with higher probabilities of cesarean delivery, newborn assisted ventilation, and antibiotic use for suspect neonatal sepsis in subsequent births. Our results are consistent with prior evidence from other health care settings that clinician practice style is sensitive to severe adverse events. More research is needed on whether these changes in clinician practice reflect overall quality and safety improvements and, in turn, improve clinical outcomes for subsequent patients.

## Notes

Supplement.
eMethods.
**eFigure.** Illustration of Stacked Difference-In-Differences Setup **eTable 1.** Covariates and Source of Data **eTable 2.** County Sample Restrictions **eTable 3.** Maternal/Birth Characteristics in Ever- vs. Never-Exposed Counties in 2011 **eTable 4.** Maternal/Birth and County Characteristics in Pre-Exposure Time by Current- vs. Future-Exposed Counties Defined Based on Different Cut-offs **eTable 5.** Maternal/Birth and County Characteristics in the Pre-Exposure Time by Current- and Future-Exposed Status Before and After Coarsened Exact Matching **eTable 6. **Post-Exposure Change in C-Section Use by Procedure Type and Low-Risk First-Birth **eTable 7. **Post-Exposure Change in Obstetric and Newborn Procedures among Current-Exposed Counties Relative to Future-Exposed Counties: Newborn Deaths in 0-2 Days **eTable 8.** Post-Exposure Change in Maternal Complications/Procedures
