# Supplementary material for: Association of Unexpected Newborn Deaths With Changes in Obstetric and Neonatal Process of Care
Source: JAMA Netw Open. 2020 Dec 7;3(12):e2024589. doi: 10.1001/jamanetworkopen.2020.24589 (PMC11902236; doi:10.1001/jamanetworkopen.2020.24589)
Supplement: Supplement. — eMethods. eFigure. Illustration of Stacked Difference-In-Differences Setup eTable 1. Covariates and Source of Data eTable 2. County Sample Restrictions eTable 3. Maternal/Birth Characteristics in Ever- vs. Never-Exposed Counties in 2011 eTable 4. Maternal/Birth and County Characteristics in Pre-Exposure Time by Current- vs. Future-Exposed Counties Defined Based on Different Cut-offs eTable 5. Maternal/Birth and County Characteristics in the Pre-Exposure Time by Current- and Future-Exposed Status Before and After Coarsened Exact Matching eTable 6. Post-Exposure Change in C-Section Use by Procedure Type and Low-Risk First-Birth eTable 7. Post-Exposure Change in Obstetric and Newborn Procedures among Current-Exposed Counties Relative to Future-Exposed Counties: Newborn Deaths in 0-2 Days eTable 8. Post-Exposure Change in Maternal Complications/Procedures [file jamanetwopen-e2024589-s001.pdf]

Supplemental Online Content

Han D, Khadka A, McConnell M, Cohen J. Association of unexpected newborn deaths with changes in obstetric and neonatal process of care. *JAMA Netw Open*. 2020;3(12):e2024589. doi:10.1001/jamanetworkopen.2020.24589

eMethods.

eFigure. Illustration of Stacked Difference-In-Differences Setup

eTable 1. Covariates and Source of Data

eTable 2. County Sample Restrictions

eTable 3. Maternal/Birth Characteristics in Ever- vs. Never-Exposed Counties in 2011

eTable 4. Maternal/Birth and County Characteristics in Pre-Exposure Time by Current- vs. Future-Exposed Counties Defined Based on Different Cut-offs

eTable 5. Maternal/Birth and County Characteristics in the Pre-Exposure Time by Current- and Future-Exposed Status Before and After Coarsened Exact Matching

eTable 6. Post-Exposure Change in C-Section Use by Procedure Type and Low-Risk First-Birth

eTable 7. Post-Exposure Change in Obstetric and Newborn Procedures among Current-Exposed Counties Relative to Future-Exposed Counties: Newborn Deaths in 0-2 Days

eTable 8. Post-Exposure Change in Maternal Complications/Procedures

This supplemental material has been provided by the authors to give readers additional information about their work.

© 2020 Han D et al. *JAMA Network Open.*

eMethods

Empirical Approach

The stacked difference-in-differences (DD) design compared "current-exposed" counties (the exposure group) that experienced a newborn death in quarter *k* to "future-exposed" counties (the comparison group) during a period when the future-exposed counties had yet to experience a death. The paired current-exposed and future-exposed counties were grouped into a DD cohort defined by *k*. All resulting DD cohorts were then stacked together for the regression analysis. eFigure 1 in this Supplement depicts how we set up each DD cohort.

The exposure and comparison groups were drawn from a total of 477 ever-exposed counties. We reported with more details how we arrived at this number in the main text. In the final analytic data, some counties were included in either the exposure or the comparison group, other counties appeared in both the exposure and the comparison group. Some future-exposed counties may appear multiple times in the comparison group if they served as comparison counties in more than one DD cohort – these would be counties with a death occurring much later in the study period. Therefore, the final count of counties in the data was larger than 477. For counties that appeared only in the comparison group, the unexpected newborn deaths occurring in these counties were not part of the 402 deaths included in the final data.

We used the future-exposed counties as the comparison group because the current-exposed and future-exposed counties were much more similar to each other across maternal and birth characteristics and birth volume than to the never-exposed counties (Table 1, eTable 3). We focused on measuring changes in the outcomes within the 9-quarter event window from *k*-4 to *k*+4, where *k* represented the quarter when the death occurred. For the comparison group, we included counties experiencing a death over the 6-quarter period (1.5 years) between *k*+4+1 and *k*+4+6. We did not go beyond *k*+4+6 because counties experiencing a death further away from *k*+4+1 were less comparable to the current-exposed counties. In eTable 4, we reported the summary statistics at the birth and county levels comparing future-exposed counties with different cutoffs to justify this decision.

Because of the way we defined the exposure (i.e., first incidence of an unexpected newborn death) and constructed our DD, counties that had higher birth volume were disproportionately represented in the exposure group relative to the comparison group. This was a limitation of our empirical approach that we could not fully address. We used Coarsened Exact Matching (CEM) to match on birth volume in an attempt to reduce the impact of the unbalanced birth volume, the post-CEM summary statistics were reported in eTable 5. We also stratified the analysis by pre-exposure birth volume (above-/below-median) and found that, while the point estimates and statistical significance of the estimates were different, the results were qualitatively consistent with our main findings (results available upon request).

In our subgroup analysis focusing on "low-risk first-birth", we defined "low-risk first-birth" as a birth following a pregnancy that was nulliparous, term, singleton, cephalic presentation, and 18-35 in maternal age, adapted from the definition used by the California Maternal Quality Care Collaborative.

Coarsened Exact Matching

We created alternative cohorts of current- and future-exposed counties using coarsened exact matching based on the pre-exposure data, that is, observations for quarter-to-event -4 through -1.[1] The matching was stratified by the DD cohorts created through the stacked DD approach, such that we matched within each cohort between counties whose first incidence of event happened in quarter *k* and their respective future-exposed comparison counties whose first incidence of event happened during the 6-quarter period [*k*+4+1, *k*+4+6].

Specifically, within each DD cohort, we created bins of data using the cut points listed below (coarsening), we then set out to find the exposure and comparison counties falling into the exact same bin for each of the matching variable (exact matching). The cut points were selected based on the distribution of our data. We focused on achieving better balance on the availability of maternal and newborn care resources and birth volume – the latter of which was the primary driver of the observed differences between the current-exposed counties and the future-exposed comparison counties arising from the way we constructed the comparison group.

**Matching covariates**	**Cut points used to coarsen data**
Number of hospitals offering obstetric services	1, 2, 3
Number of hospitals with neonatal intensive care unit (NICU)	1
Number of obstetricians per 1,000 women aged 15-44 years	0.5
Number of average quarterly births	10th through 90th deciles

eTable 5 in this Supplement reported the summary statistics before and after CEM. The “balance” between the current- and future- exposed counties improved somewhat after CEM across many maternal/birth and county characteristics but particularly quarterly birth volume, NICU availability, and rural/urban status. There were still some discrepancies between the two groups, which was expected given the way we constructed the comparison group and because we only matched on four variables.

Reference

1. Iacus SM, King G, Porro G. Causal Inference Without Balance Checking: Coarsened Exact Matching. *Polit Anal*. 2012;20(1):1--24.

© 2020 Han D et al. *JAMA Network Open.*

eFigure 1. Illustration of Stacked Difference-In-Differences Setup [a]

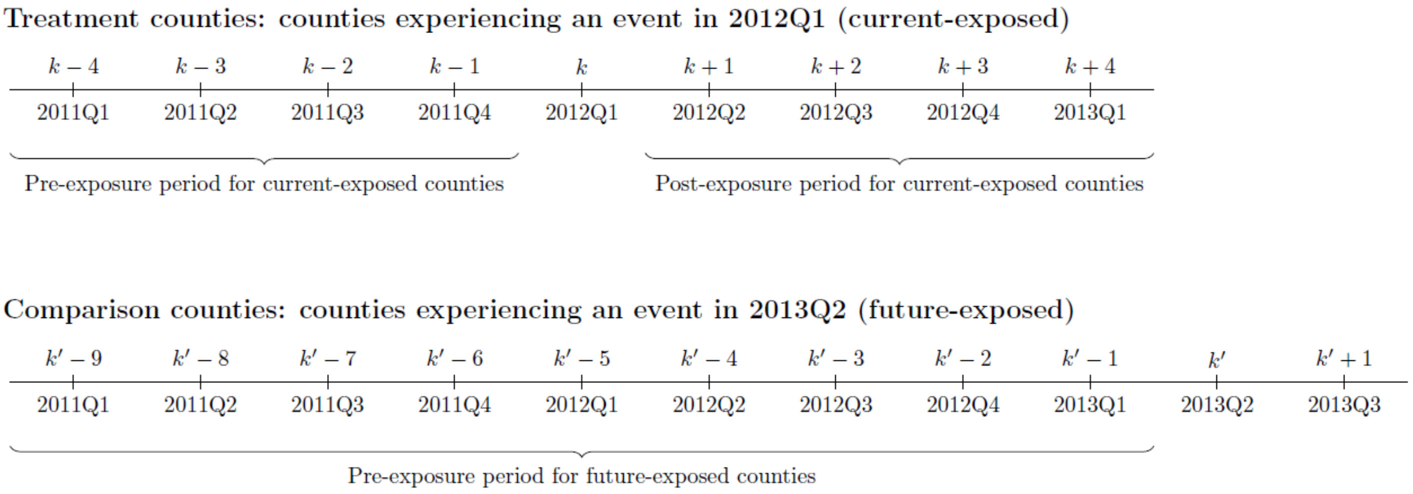

a. Stacked difference-in-differences uses the pre-exposure period for future-exposed counties as comparison for the current-exposed counties.

© 2020 Han D et al. *JAMA Network Open.*

eTable 1. Covariates and Source of Data

Covariate definition and categories	Source of data
Maternal and pregnancy characteristics	
Race/ethnicity: White, Black, Hispanic	Vital statistics' Natality files
Marital status	
Maternal age	
Mother's highest education: less than high school, high school or some college, college degree and above	
Nulliparous or multiparous pregnancy	
Singleton or multiple pregnancy	
Health insurance coverage: Medicaid, private insurance, uninsured	
Smoking status during pregnancy	
Late or no antenatal care: starting antenatal care in the 3rd trimester or reporting no antenatal care	
Maternal infection: the presence of any of gonorrhea, syphilis, chlamydia, hepatitis B, hepatitis C	
Obesity: BMI ≥ 35	
History of C-section	
Pre-pregnancy or gestational diabetes	
Pre-pregnancy or gestational hypertension	
Preterm birth: gestational age < 37 weeks	
Breech presentation	
Sex of the infant	
Birthweight < 2,500 grams	
Birthweight > 4,000 grams	
Congenital anomalies diagnosed prenatally or after delivery: the presence of any of anencephaly, meningomyelocele/spina bifida, cyanotic congenital heart disease, congenital diaphragmatic hernia, omphalocele, gastroschisis, limb reduction defect, cleft lip w/ or w/o left palate, cleft palate alone, down syndrome, suspected chromosomal disorder, hypospadias	
County-level characteristics	
Rural/urban status	NCHS Urban-Rural Classification Scheme
Median household income	Small Area Income and Poverty Estimates
Poverty rate	Small Area Income and Poverty Estimates
Uninsured rate among individuals aged 18-64 years	Small Area Health Insurance Estimates
Number of hospitals offering obstetric services	CMS Provider of Services File
Number of hospitals with neonatal intensive care unit	CMS Provider of Services File

© 2020 Han D et al. *JAMA Network Open.*

Covariate definition and categories	Source of data
Number of obstetricians per 1,000 women aged 15-44 years	National Plan and Provider Enumeration System, Census county population estimates
Number of primary care physicians per 1,000 individuals	National Plan and Provider Enumeration System, Census county population estimates

© 2020 Han D et al. *JAMA Network Open.*

eTable 2. County Sample Restrictions

**Sample restrictions**	**Remaining counties (unique count)**
All counties in the data	2,373
Drop states not using revised birth certificate	1,919
Drop never-exposed counties	737
Drop counties whose first incidence of death occurred before 2012Q1	517
Drop counties whose number of average quarterly birth was less than 25 or with fewer than 25 births in any single quarter	479
Drop counties with more than 10 deaths	477

© 2020 Han D et al. *JAMA Network Open.*

eTable 3. Maternal/Birth Characteristics in Ever- vs. Never-Exposed Counties in 2011 [a, b, c]

Characteristics		No. (%) Total (N = 1,035,871)	Ever-exposed counties (N = 807,811)	Never-exposed counties (N = 228,060)
Race/ethnicity				
	White	687,003 (66.3)	525,429 (65.0)	161,574 (70.8)
	Black	119,871 (11.6)	103,712 (12.8)	16,159 (7.1)
	Hispanic	185,553 (17.9)	142,055 (17.6)	43,498 (19.1)
	Other	43,444 (4.2)	36,615 (4.5)	6,829 (3.0)
Insurance coverage [d]				
	Medicaid	476,799 (46.0)	357,881 (44.3)	118,918 (52.1)
	Private insurance	479,327 (46.3)	391,096 (48.4)	88,231 (38.7)
	Uninsured	34,486 (3.3)	23,932 (3.0)	10,554 (4.6)
Highest education				
	Less than high school	183,849 (17.7)	137,438 (17.0)	46,411 (20.4)
	High school or some college	588,255 (56.8)	446,639 (55.3)	141,616 (62.1)
	College degree or above	263,767 (25.5)	223,734 (27.7)	40,033 (17.6)
Maternal age, Mean (SD)		27.2 (5.9)	27.5 (5.9)	26.2 (5.7)
Married		609,758 (58.9)	485,128 (60.1)	124,630 (54.6)
Nulliparous		336,599 (32.5)	262,900 (32.5)	73,699 (32.3)
Multiple gestations		32,364 (3.1)	27,384 (3.4)	4,980 (2.2)
Smoking during pregnancy		136,439 (13.2)	94,680 (11.7)	41,759 (18.3)
Late ANC or no ANC		58,676 (5.7)	45,036 (5.6)	13,640 (6.0)
Obese		255,714 (24.7)	194,325 (24.1)	61,389 (26.9)
Maternal infection		27,620 (2.7)	21,059 (2.6)	6,561 (2.9)
Chronic/gestational diabetes		61,386 (5.9)	48,640 (6.0)	12,746 (5.6)
Chronic/gestational hypertension		67,084 (6.5)	53,527 (6.6)	13,557 (5.9)
Previous C-section		147,116 (14.2)	115,062 (14.2)	32,054 (14.1)
Breech presentation		37,298 (3.6)	30,019 (3.7)	7,279 (3.2)
Birthweight, Mean (SD)		3,286.9 (571.1)	3,280.8 (583.1)	3,308.4 (525.7)
Preterm birth		113,455 (11.0)	91,591 (11.3)	21,864 (9.6)
Female infant		505,675 (48.8)	394,608 (48.8)	111,067 (48.7)
Congenital anomalies		3,996 (0.4)	3,119 (0.4)	877 (0.4)
Mode of delivery: C-section		334,119 (32.3)	260,019 (32.2)	74,100 (32.5)

a. Since pre- and post-exposure times were not defined for never-exposed group, the data shown here included data for year 2011.
b. Exclude 14 states using the old birth certificate: Alaska, Alabama, Arkansas, Arizona, Connecticut, Hawaii, Massachusetts, Maine, Minnesota, Mississippi, New Jersey, Rhode Island, Virginia, and West Virginia.
c. SD = standard deviation
d. Numbers do not add up to 100% due to other/unknown insurance type.

© 2020 Han D et al. *JAMA Network Open.*

eTable 4. Maternal/Birth and County Characteristics in Pre-Exposure Time by Current- vs. Future-Exposed Counties Defined Based on Different Cut-offs [a, b, c]

		Current-exposed counties	Future-exposed counties with an event in quarter		
			[k+4+1, k+4+6] (our specification)	[k+4+7, k+4+8]	>= k+4+9
**Panel A: Maternal/Birth Characteristics**		**N = 840,518**	**N = 1,698,513**	**N = 322,059**	**N = 719,431**
		**No. (%)**			
Race/ethnicity					
	White	540,456 (64.3)	1,160,076 (68.3)	231,343 (71.8)	530,265 (73.7)
	Black	113,638 (13.5)	192,074 (11.3)	32,663 (10.1)	72,244 (10.0)
	Hispanic	147,299 (17.5)	272,619 (16.1)	45,366 (14.1)	88,319 (12.3)
	Other	39,125 (4.7)	73,744 (4.3)	12,687 (3.9)	28,603 (4.0)
Insurance coverage [d]					
	Medicaid	371,953 (44.3)	832,927 (49.0)	156,372 (48.6)	369,103 (51.3)
	Private insurance	405,371 (48.2)	735,030 (43.3)	139,370 (43.3)	299,021 (41.6)
	Uninsured	24,083 (2.9)	50,461 (3.0)	9,156 (2.8)	20,615 (2.9)
Highest education					
	Less than high school	134,187 (16.0)	288,507 (17.0)	53,006 (16.5)	123,280 (17.1)
	High school or some college	472,170 (56.2)	1,013,764 (59.7)	194,819 (60.5)	438,763 (61.0)
	College degree or above	234,161 (27.9)	396,242 (23.3)	74,234 (23.0)	157,388 (21.9)
Maternal age, Mean (SD)		27.6 (5.8)	27.2 (5.8)	27.0 (5.8)	26.7 (5.7)
Married		500,471 (59.5)	961,977 (56.6)	183,497 (57.0)	403,730 (56.1)
Nulliparous		269,432 (32.1)	547,432 (32.2)	103,702 (32.2)	236,046 (32.8)
Multiple gestations		28,483 (3.4)	48,068 (2.8)	8,804 (2.7)	19,596 (2.7)
Smoking during pregnancy		95,846 (11.4)	232,304 (13.7)	48,564 (15.1)	120,577 (16.8)
Late ANC or no ANC		47,563 (5.7)	96,930 (5.7)	16,014 (5.0)	36,988 (5.1)
Obese		206,615 (24.6)	443,357 (26.1)	85,921 (26.7)	190,099 (26.4)
Maternal infection		23,521 (2.8)	46,663 (2.7)	9,088 (2.8)	20,726 (2.9)
Chronic/gestational diabetes		51,321 (6.1)	101,749 (6.0)	18,682 (5.8)	39,971 (5.6)
Chronic/gestational hypertension		58,658 (7.0)	112,363 (6.6)	20,988 (6.5)	49,476 (6.9)
Previous C-section		121,190 (14.4)	245,662 (14.5)	47,076 (14.6)	101,586 (14.1)
Breech presentation		31,197 (3.7)	60,089 (3.5)	11,311 (3.5)	24,961 (3.5)
Birthweight, Mean (SD)		3,281.8 (585.4)	3,298.6 (559.7)	3,307.1 (549.1)	3,292.9 (558.5)
Preterm birth		94,854 (11.3)	176,321 (10.4)	32,226 (10.0)	76,629 (10.7)
Female infant		409,997 (48.8)	828,721 (48.8)	157,233 (48.8)	352,716 (49.0)
Congenital anomalies		3,193 (0.4)	5,817 (0.3)	1,211 (0.4)	2,830 (0.4)
Method of delivery: C-section		269,678 (32.1)	547,197 (32.2)	103,459 (32.1)	228,412 (31.7)
**Panel B: County Characteristics (county-year measures)**		**N = 659** [e]	**N = 2,405** [e]	**N = 544** [e]	**N = 1,346** [e]
Births per quarter [f]		589.9 (717.6)	351.2 (416.0)	294.4 (278.7)	291.7 (235.7)
Population (000s)		178.7 (206.1)	115.7 (143.0)	98.2 (96.9)	92.0 (70.6)

© 2020 Han D et al. *JAMA Network Open.*

		Current-exposed counties	Future-exposed counties with an event in quarter		
			[k+4+1, k+4+6] (our specification)	[k+4+7, k+4+8]	>= k+4+9
Non-metropolitan (%)		40.4 (49.1)	46.7 (49.9)	52.0 (50.0)	50.3 (50.0)
Number of hospitals with obstetric services		1.8 (1.3)	1.5 (0.9)	1.4 (0.8)	1.3 (0.7)
Percent counties without NICU		64.0 (48.0)	75.7 (42.9)	81.4 (38.9)	81.5 (38.8)
OB/GYN per 1,000 female aged 15-44 years		0.6 (0.5)	0.5 (0.3)	0.5 (0.4)	0.5 (0.4)
Primary care physicians per 1,000 individuals		1.0 (0.8)	0.9 (0.5)	0.9 (0.5)	0.9 (0.5)
Median household income (2017 $)		52.0 (13.3)	49.8 (11.5)	48.9 (10.6)	48.5 (9.6)
Percent population in poverty		16.2 (5.6)	16.7 (5.7)	17.0 (5.6)	17.2 (5.7)
Uninsured rate among individuals aged 18-64 years		16.0 (5.3)	15.8 (5.4)	16.3 (5.4)	16.7 (5.2)

a. Included data for quarter-to-event -4 through -1.
b. Exclude 14 states using the old birth certificate: Alaska, Alabama, Arkansas, Arizona, Connecticut, Hawaii, Massachusetts, Maine, Minnesota, Mississippi, New Jersey, Rhode Island, Virginia, and West Virginia.
c. SD = standard deviation; NICU = neonatal intensive care unit; OB/GYN = obstetrician/gynecologist.
d. Numbers do not add up to 100% due to other/unknown insurance type.
e. The N for county characteristics in Panel B is the number of county-year observations. It is larger than the number of individual counties because the quarter-to-event time did not necessarily align with the calendar year, so some partial year data was used.
f. Quarterly birth volume averaging over the 1-year pre-exposure period (quarter-to-event -4 through -1).

© 2020 Han D et al. *JAMA Network Open.*

eTable 5. Maternal/Birth and County Characteristics in the Pre-Exposure Time by Current- and Future-Exposed Status Before and After Coarsened Exact Matching [a, b, c, d]

	Pre-CEM		Post-CEM	
	**Current-exposed**	**Future-exposed**	**Current-exposed**	**Future-exposed**
**Panel A: Maternal/Birth Characteristics**	**N = 840,518**	**N = 1,698,513**	**N = 572,943**	**N = 942,494**
	**No. (%)**			
Race/ethnicity				
White	540,456 (64.3)	1,160,076 (68.3)	366,376 (63.9)	631,944 (67.1)
Black	113,638 (13.5)	192,074 (11.3)	80,181 (14.0)	104,436 (11.1)
Hispanic	147,299 (17.5)	272,619 (16.1)	100,916 (17.6)	164,196 (17.4)
Other	39,125 (4.7)	73,744 (4.3)	25,470 (4.4)	41,918 (4.4)
Insurance coverage [e]				
Medicaid	371,953 (44.3)	832,927 (49.0)	259,567 (45.3)	459,894 (48.8)
Private insurance	405,371 (48.2)	735,030 (43.3)	271,176 (47.3)	404,397 (42.9)
Uninsured	24,083 (2.9)	50,461 (3.0)	17,902 (3.1)	28,706 (3.0)
Highest education				
Less than high school	134,187 (16.0)	288,507 (17.0)	96,267 (16.8)	167,751 (17.8)
High school or some college	472,170 (56.2)	1,013,764 (59.7)	323,786 (56.5)	553,725 (58.8)
College degree or above	234,161 (27.9)	396,242 (23.3)	152,890 (26.7)	221,018 (23.5)
Maternal age, Mean (SD)	27.6 (5.8)	27.2 (5.8)	27.4 (5.8)	27.2 (5.9)
Married	500,471 (59.5)	961,977 (56.6)	336,404 (58.7)	538,132 (57.1)
Obese	269,432 (32.1)	547,432 (32.2)	182,420 (31.8)	303,796 (32.2)
Smoking during pregnancy	28,483 (3.4)	48,068 (2.8)	19,071 (3.3)	27,164 (2.9)
Maternal infection	95,846 (11.4)	232,304 (13.7)	68,720 (12.0)	125,651 (13.3)
Late ANC or no ANC	47,563 (5.7)	96,930 (5.7)	33,153 (5.8)	56,599 (6.0)
Nulliparous	206,615 (24.6)	443,357 (26.1)	143,243 (25.0)	243,520 (25.8)
Multiple gestations	23,521 (2.8)	46,663 (2.7)	17,035 (3.0)	26,100 (2.8)
Chronic/gestational diabetes	51,321 (6.1)	101,749 (6.0)	34,973 (6.1)	57,415 (6.1)
Chronic/gestations hypertension	58,658 (7.0)	112,363 (6.6)	40,571 (7.1)	62,008 (6.6)
Previous C-section	121,190 (14.4)	245,662 (14.5)	82,067 (14.3)	136,775 (14.5)
Breech presentation	31,197 (3.7)	60,089 (3.5)	21,160 (3.7)	33,371 (3.5)
Birth weight, Mean (SD)	3,281.8 (585.3)	3,298.6 (559.7)	3,279.8 (584.6)	3,294.4 (561.6)
Preterm birth	94,854 (11.3)	176,321 (10.4)	64,895 (11.3)	99,392 (10.5)
Congenital anomalies	409,997 (48.8)	828,721 (48.8)	279,513 (48.8)	459,876 (48.8)
Female infant	3,193 (0.4)	5,817 (0.3)	2,221 (0.4)	3,185 (0.3)
Mode of this delivery: C-section	269,678 (32.1)	547,197 (32.2)	180,728 (31.5)	307,522 (32.6)
**Panel B: County characteristics (county-year measures)**	**N = 659 [f]**	**N = 2,405 [f]**	**N = 521 [f]**	**N = 1,298 [f]**
	**Mean (SD)**			
Births per quarter [g]	589.9 (717.6)	351.2 (416.0)	502.8 (671.1)	344.4 (473.3)
Population (000s)	178.7 (206.1)	115.7 (143.0)	147.6 (173.3)	114.8 (162.9)

© 2020 Han D et al. *JAMA Network Open.*

Non-metropolitan (%)	40.4 (49.1)	46.7 (49.9)		46.3 (49.9)	49.4 (50.0)
**Characteristics**	**Current exposed**	**Future exposed**		**Current exposed**	**Future exposed**
Number of hospitals with obstetric services	1.8 (1.3)	1.5 (0.9)		1.6 (1.2)	1.4 (0.9)
Percent counties without NICU	64.0 (48.0)	75.7 (42.9)		70.2 (45.8)	76.8 (42.2)
OB/GYN per 1,000 female aged 15-44 years	0.6 (0.5)	0.5 (0.3)		0.6 (0.5)	0.5 (0.3)
Primary care physicians per 1,000 individuals	1.0 (0.8)	0.9 (0.5)		1.0 (0.8)	0.9 (0.5)
Median household income (2017 $)	52.0 (13.3)	49.8 (11.5)		51.1 (12.6)	49.8 (11.7)
Percent population in poverty	16.2 (5.6)	16.7 (5.7)		16.3 (5.4)	16.6 (5.5)
Uninsured rate among individuals aged 18-64 years	16.0 (5.3)	15.8 (5.4)		16.1 (5.2)	16.2 (5.3)

a. Included data for quarter-to-event -4 through -1.
b. Excluded 14 states using the old birth certificate: Alaska, Alabama, Arkansas, Arizona, Connecticut, Hawaii, Massachusetts, Maine, Minnesota, Mississippi, New Jersey, Rhode Island, Virginia, and West Virginia
c. Coarsened Exact Matching was conducted using four variables: number of hospitals offering obstetric services; number of hospitals with NICUs; number of obstetricians per 1,000 women aged 15-44 years; and number of average quarterly births. Pre-CEM refers to the sample prior to Coarsened Exact Matching; Post-CEM refers to the sample after Coarsened Exact Matching
d. CEM = Coarsened Exact Matching; SD = standard deviation; NICU = neonatal intensive care unit; OB/GYN = obstetrician/gynecologist.
e. Numbers do not add up to 100% due to other/unknown insurance type.
f. The N for county characteristics in Panel B is the number of county-year observations. It is larger than the number of individual counties because the quarter-to-event time did not necessarily align with the calendar year, so some partial year data was used.
g. Quarterly birth volume averaging over the 1-year pre-exposure period (quarter-to-event -4 through -1).

© 2020 Han D et al. *JAMA Network Open.*

eTable 6. Post-Exposure Change in C-Section Use by Procedure Type and Low-Risk First-Birth [a, b, c, d]

	C-section	Planned C-section	Unplanned C-section
Panel A-1: Low-Risk First-Births			
Point Estimate	0.60[e]	0.24	0.34
95% CI	[0.07, 1.14]	[-0.22, 0.69]	[-0.24, 0.93]
P-value	0.03	0.31	0.25
Mean of Dep. Variable	25.46	7.30	18.02
Observations	1,417,810	1,415,157	1,415,157
Panel A-2: Other Births			
Point Estimate	0.14	0.00	0.12
95% CI	[-0.18, 0.46]	[-0.35, 0.36]	[-0.15, 0.40]
P-value	0.39	0.98	0.38
Mean of Dep. Variable	34.36	27.53	6.67
Observations	4,305,391	4,294,953	4,294,953

a. Excluded 14 states using the old birth certificate: Alaska, Alabama, Arkansas, Arizona, Connecticut, Hawaii, Massachusetts, Maine, Minnesota, Mississippi, New Jersey, Rhode Island, Virginia, and West Virginia.
b. All coefficients were estimated using linear regressions with a binary dependent variable and thus represent percentage point changes in the outcome.
c. 95% CI = 95% confidence intervals. All confidence intervals were calculated using standard errors clustered at the county level.
d. Low-risk first-births included term, singleton, and nulliparous pregnancies with cephalic presentation in women aged 18-35 years.
e. Statistically significant after controlling for a false positive rate of 0.1 using the Benjamini-Hochberg procedure.

© 2020 Han D et al. *JAMA Network Open.*

eTable 7. Post-Exposure Change in Obstetric and Newborn Procedures among Current-Exposed Counties Relative to Future-Exposed Counties: Newborn Deaths in 0-2 Days [a, b, c, d]

	Induction	C-section	Forceps or vacuum	Augmentation	Assisted ventilation (immediate or > 6 hours)	Surfactant replacement therapy	Antibiotics for suspected neonatal sepsis	Admission to NICU
Point Estimate	-0.54	0.15	0.05	0.51	0.25	0.04	0.30 [e]	0.33 [e]
95% CI	[-1.62, 0.55]	[-0.13, 0.44]	[-0.09, 0.20]	[-0.36, 1.38]	[-0.06, 0.56]	[-0.03, 0.11]	[0.10, 0.51]	[0.05, 0.61]
P-value	0.33	0.28	0.49	0.25	0.11	0.29	<0.01	0.02
Mean of Dep. Variable	26.20	32.39	3.36	22.16	3.70	0.50	2.42	7.87
Observations	8,094,577	8,094,966	8,094,973	8,094,577	8,083,890	8,083,890	8,083,890	8,083,890

a. Excluded 14 states using the old birth certificate: Alaska, Alabama, Arkansas, Arizona, Connecticut, Hawaii, Massachusetts, Maine, Minnesota, Mississippi, New Jersey, Rhode Island, Virginia, and West Virginia.
b. All coefficients were estimated using linear regressions with a binary dependent variable and thus represent percentage point changes in the outcome.
c. NICU = neonatal intensive care unit; 95% CI = 95% confidence intervals. All confidence intervals were calculated using standard errors clustered at the county level.
d. The analysis using newborn deaths in 0-2 days was not a subgroup analysis. The "event" variable was created based on this alternative definition and a new analytic dataset was constructed following the same data processing used for constructing the main analytic sample.
e. Statistically significant after controlling for a false positive rate of 0.1 using the Benjamini-Hochberg procedure within each of the two domains – delivery-related procedures (induction, C-section, forceps/vacuum, augmentation) and newborn procedures (assisted ventilation, surfactant therapy, antibiotics use, NICU admission).

© 2020 Han D et al. *JAMA Network Open.*

eTable 8. Post-Exposure Change in Maternal Complications/Procedures [a, b, c]

	Maternal transfusion	3rd/4th degree perineal laceration	Unplanned hysterectomy	Ruptured uterus	Admission to ICU
Panel A: Full Set of Counties					
Point Estimate	-0.01	-0.11	-0.01	0.00	0.01
95% CI	[-0.04, 0.03]	[-0.26, 0.05]	[-0.02, 0.00]	[-0.01, 0.00]	[-0.01, 0.03]
P-value	0.76	0.17	0.11	0.40	0.48
Mean of Dep. Variable	0.32	1.05	0.04	0.02	0.12
Observations	5,717,023	5,717,023	5,717,023	5,717,023	5,717,023
Panel B: Counties with 1 Hospital					
Point Estimate	0.04	-0.26	-0.02	0.01	0.02
95% CI	[-0.02, 0.11]	[-0.72, 0.20]	[-0.03, 0.00]	[-0.01, 0.03]	[-0.01, 0.06]
P-value	0.18	0.27	0.11	0.16	0.17
Mean of Dep. Variable	0.38	1.03	0.04	0.03	0.11
Observations	1,862,675	1,862,675	1,862,675	1,862,675	1,862,675
Panel C: Full Set of Counties, Coarsened Exact Matching					
Point Estimate	-0.02	-0.14	-0.02 [d]	-0.00	0.01
95% CI	[-0.06, 0.02]	[-0.32, 0.04]	[-0.03, -0.01]	[-0.01, 0.01]	[-0.02, 0.03]
P-value	0.27	0.12	<0.01	0.76	0.58
Mean of Dep. Variable	0.31	1.12	0.04	0.02	0.13
Observations	3,425,352	3,425,352	3,425,352	3,425,352	3,425,352
Panel D: Counties with 1 Hospital, Coarsened Exact Matching					
Point Estimate	0.05	-0.21	-0.01	0.01	0.02
95% CI	[-0.02, 0.11]	[-0.67, 0.26]	[-0.03, 0.01]	[-0.01, 0.03]	[-0.01, 0.06]
P-value	0.18	0.38	0.19	0.24	0.25
Mean of Dep. Variable	0.34	1.01	0.04	0.02	0.11
Observations	1,281,470	1,281,470	1,281,470	1,281,470	1,281,470

a. Excluded 14 states using the old birth certificate: Alaska, Alabama, Arkansas, Arizona, Connecticut, Hawaii, Massachusetts, Maine, Minnesota, Mississippi, New Jersey, Rhode Island, Virginia, and West Virginia.
b. All coefficients were estimated using linear regressions with a binary dependent variable and thus represent percentage point changes in the outcome.
c. ICU = intensive care unit; 95% CI = 95% confidence intervals. All confidence intervals were calculated using standard errors clustered at the county level.
d. Statistically significant after controlling for a false positive rate of 0.1 using the Benjamini-Hochberg procedure.

© 2020 Han D et al. *JAMA Network Open.*
